# Matrix Metalloproteinases Polymorphisms as Prognostic Biomarkers in Malignant Pleural Mesothelioma

**DOI:** 10.1155/2017/8069529

**Published:** 2017-09-12

**Authors:** Danijela Štrbac, Katja Goričar, Vita Dolžan, Viljem Kovač

**Affiliations:** ^1^Institute of Oncology Ljubljana, Ljubljana, Slovenia; ^2^Pharmacogenetics Laboratory, Institute of Biochemistry, Faculty of Medicine, University of Ljubljana, Ljubljana, Slovenia

## Abstract

**Background:**

Malignant pleural mesothelioma (MPM) is a rare disease with a relatively short overall survival (OS). Metalloproteinases (MMPs) have a vast biological effect on tumor progression, invasion, metastasis formation, and apoptosis. MMP expression was previously associated with survival in MPM. Our aim was to evaluate if genetic variability of *MMP* genes could also serve as a prognostic biomarker in MPM.

**Methods:**

We genotyped 199 MPM patients for ten polymorphisms: rs243865, rs243849 and rs7201, in *MMP2;* rs17576, rs17577, rs20544, and rs2250889 in *MMP9*; and rs1042703, rs1042704, and rs743257 in *MMP14*. We determined the influence on survival using Cox regression.

**Results:**

Carriers of polymorphic *MMP9* rs2250889 allele had shorter time to progression (TTP) (6.07 versus 10.03 months, HR = 2.45, 95% CI = 1.45–4.14, *p* = 0.001) and OS (9.23 versus 19.2 months, HR = 2.39, 95% CI = 1.37–4.18, *p* = 0.002). In contrast, carriers of at least one polymorphic *MMP9* rs20544 allele had longer TTP (10.93 versus 9.40 months, HR = 0.57, 95% CI = 0.38–0.86 *p* = 0.007) and OS (20.67 versus 13.50 months, HR = 0.56, 95% CI = 0.37–0.85, *p* = 0.007). *MMP14* rs1042703 was associated with nominally shorter TTP (8.7 versus 9.27 months, HR = 2.09, 95% CI = 1.06–4.12, *p* = 0.032).

**Conclusions:**

Selected *MMP* SNPs were associated with survival and could be used as potential genetic biomarkers in MPM.

## 1. Introduction

Malignant pleural mesothelioma (MPM) is a rare disease, linked to asbestos exposure in more than 80% of the cases. The latency period can last up to thirty years and estimated median survival is from 9–12 months. The worldwide incidence of mesothelioma is approximately 94,000 cases per year. The incidence of mesothelioma is rising worldwide, with the most affected areas being Europe, Australia, and the USA [1]. The Slovenian national registry follows the data on mesothelioma since 1961, and the current incidence is about 43 new cases per year in a population of roughly 2 million [2].

Over the last decade, the standard treatment of mesothelioma has not changed. It relies on surgery, chemotherapy, and radiation-based approaches [3]. In Slovenia, chemotherapy with cisplatin doublets became a standard part of multimodal treatment in 2003. This led to improved median survival of 13.6 months, as reported in a population-based survey of 444 Slovenian MPM patients [4]. The influence of platinum pathway and folate pathway polymorphisms on treatment outcome and toxicity has been studied extensively in the Slovenian MPM population [5, 6]. The most recent study proposes an algorithm based on clinical-pharmacogenetic models for stratification of MPM patients and personalized cancer treatment [7]. Newer treatment options, including targeted treatments and immunotherapy, are being researched and implemented in clinical trials [8, 9], with the aim of further improving treatment outcome in MPM. Although several clinical (gender, age, ECOG performance status…), and genetic (chromosomal alterations, DNA methylation, and microRNA expression) factors were reported to be associated with mechanisms linked to risk for MPM and/or its progression, better biomarkers that could help predict survival of these patients are needed [10, 11].

Recent studies have identified matrix metalloproteinases (MMPs) as modulators of the tumor microenvironment with an important role in carcinogenesis [12]. MMPs are calcium-dependent, zinc-containing endopeptidases, with three common domains containing the propeptide, catalytic, and heamopexin-like terminal domain [13]. They are involved in tissue remodeling by interfering with the cell-cell and cell-extracellular matrix interactions. Studies have shown that MMPs, particularly MMP-2 and MMP-9, play a role in tumor angiogenesis, invasion, and metastases [14]. The studies performed thus far show that MMPs and their inhibitory molecules, tissue inhibitors of metalloproteinases (TIMPs) have an important role in proliferation and progression of MPM and other malignancies. Different MMPs (MMP2, MMP9, MMP11, and MMP14) and their expression were studied in the mesothelioma tissue, but only a few have been prognostically significant. While the increasing MMP2 and pro-MMP2 concentrations were independently associated with a poor prognosis, MMP9 concentration had no prognostic significance [15]. In another study, only a small sample of 49 patients was analyzed with the conclusion that MMP2 and MMP9 overexpression might be related to tumor kinetics but warrants further investigation [16]. High MMP14 expression was proven to have a prognostic value, influencing overall survival (OS) in a larger cohort of MPM patients. The calculated relative risk of death in MPM patients with low *MMP14* expression was significantly lower than in patients with high *MMP14* expression. It is therefore not surprising that MMP14 molecule has also been proposed as a potential therapeutic target in MPM [17].

Common genetic polymorphisms that may influence MMP expression levels (as well as cancer risk) have been reported in all genes coding for the abovementioned MMPs. Genetic polymorphisms in *MMPs* have been studied in other more frequent malignancies, such as breast, rectal, and prostate cancer [18, 19], and some of them were proposed as prognostic biomarkers in different cancers [20, 21]. Keeping in mind the potential role of MMPs in mesothelioma and considering that only expression of MMP2, MMP9, and MMP14 has been studied in mesothelioma, we set out to perform a study exploring genetic variability of these genes. Our aim was to analyze common putative functional single nucleotide polymorphisms (SNPs) in *MMP2*, *MMP9*, and *MMP14* genes and to evaluate these SNPs as potential prognostic genetic biomarkers in MPM.

## 2. Patients and Methods

### 2.1. Patients

Patients with histologically proven pleural or peritoneal mesothelioma diagnosed and treated between 2007 and 2015 were included in this retrospective study. Patients were diagnosed mostly at the University Clinic of Golnik and University Clinical Center Ljubljana, Department of Thoracic Surgery. Most of the patients were treated and followed up at the Institute of Oncology, Ljubljana.

Most patients included in the study were also participating in previous studies on pharmacogenomics of MPM treatment at the Institute of Oncology, Ljubljana. Some of the patients were included in a parallel clinical trial AGILI (Trial registration ID NCT01281800). All of the patients were included consecutively. The majority of patients had a performance status of 0–2 (ECOG), since they were the ones receiving chemotherapy. However, elderly patients with ECOG performance status of 3, considered only for best supportive care, were also included. Due to the rarity of the disease and the size of the general population, no additional selection criteria were used.

The study was approved by the Slovenian Ethics Committee for Research in Medicine and was carried out according to the Declaration of Helsinki.

### 2.2. End Points of the Study

Considering the retrospective nature of the study, time to progression (TTP) was chosen as an end point as well as overall survival (OS). TTP was defined as time from diagnosis to progression, and OS was defined as time from diagnosis to death of any cause. The patients that did not progress or die at the time of analysis were censored at the time of the last follow-up. Progression was assessed radiologically, using at least a chest X-ray; however, the majority of patients had either a CT scan or a PET/CT scan.

### 2.3. DNA Extraction and Genotyping

Genomic DNA was extracted from whole-blood frozen samples collected at the inclusion in any of the abovementioned studies using the Qiagen FlexiGene Kit (Qiagen, Hilden, Germany) in accordance with the manufacturer's instructions.

Putatively functional SNPs with minor allele frequency of at least 5% in European population were selected for analysis: all nonsynonymous SNPs and SNPs in 3′ and 5′ untranslated regions. No SNPs in intronic regions were selected. Additionally, some SNPs were selected based on previously published literature. Ten different polymorphisms in three *MMP* genes fulfilling these criteria were genotyped: *MMP2* rs243865, rs243849, and rs7201; *MMP9* rs17576, rs17577, rs2250889, and rs20544; and *MMP14* rs1042703, rs1042704, and rs743257. Predicted function of these polymorphisms was assessed using SNP function prediction [22]. For SNPs in 5′ or 3′ untranslated regions, HaploReg v4.1 [23] and GTEx [24] were also used.

The genotyping of all the SNPs was carried out using a fluorescence-based competitive allele-specific assay (KASPar), according to the manufacturer's instructions (LGC Genomics, UK). For all investigated polymorphisms, 15% of samples were genotyped in duplicates. Genotyping quality control criteria included 100% duplicate call rate and 95% SNP-wise call rate.

### 2.4. Statistical Analyses

Continuous and categorical variables were described using median and range (25%–75%) and frequencies, respectively. Deviation from the Hardy-Weinberg equilibrium (HWE) was assessed using the standard chi-square test. The additive and dominant genetic model was used in statistical analyses. The influence of genetic polymorphism on TTP and OS was examined by Cox regression to calculate hazard ratios (HRs) and their 95% confidence intervals (CIs). Clinical variables used for adjustment in multivariable survival analysis were selected from clinical variables at diagnosis using stepwise forward conditional selection.

All statistical analyses were carried out by Statistical Package for the Social Sciences (SPSS) for Windows, version 21.0 (IBM Corporation, Armonk, NY, USA). Haplotypes were reconstructed and analyzed using THESIAS software. The most frequent haplotype was used as the reference. All statistical tests were two-sided. To reduce the chance of false positive results, multiple testing analysis by false discovery rate from the Genetic Type I error calculator was used to select the threshold for *p* values [25]. *p* values up to 0.01 were considered statistically significant, while *p* values between 0.01 and 0.05 were considered nominally significant.

## 3. Results

### 3.1. Patient Characteristics

In total, we included 199 patients with MPM. Clinical characteristics of the study group are summarized in Table 1. To the date of analyses, the median TTP was 7.67 (5.27–13.80) months with the median OS of 16.3 (9.07–26.80) months and a long follow-up of 69.67 (22.00–81.53) months.

### 3.2. Genotyping Analysis

Genotype frequencies of investigated SNPs and their predicted functions are presented in Table 2. The distributions of all the investigated SNPs were in agreement with the HW equilibrium. Duplicate call rate was 100% for all SNPs. SNP-wise call rate was 100% for six SNPs, 99.5% for one SNP, 99.0% for two SNPs, and 97.5% for one SNP. The number of missing genotypes is presented in Table 2. One patient had two missing genotypes, and eight had one missing genotype; for the rest, genotype information was complete.

### 3.3. Time to Progression Analysis

The results of TTP analysis are shown in Table 3 (analysis adjusted for clinical variables) and Supplementary Table 1 available online at https://doi.org/10.1155/2017/8069529 (unadjusted analysis). In multivariable analysis, histological type, weight loss, and performance status were significantly associated with TTP. Carriers of polymorphic *MMP9* rs2250889 allele had shorter TTP (6.07 versus 10.03 months, *p* = 0.001, HR = 2.45, 95% CI = 1.45–4.14) compared to noncarriers. These results remained significant also after adjustment for histological type, weight loss, and performance status (HR = 2.32, 95% CI = 1.34–4.03, *p* = 0.003).

On the other hand, carriers of at least one polymorphic *MMP9* rs20544 allele had longer TTP than noncarriers (10.13 versus 7.53 months, *p* = 0.015, HR = 0.63, 95% CI = 0.43–0.91). The association remained nominally significant after adjustment for histological type, weight loss, and performance status (HR = 0.62, 95% CI = 0.43–0.92, *p* = 0.016).

A nominally significant association with shorter TTP was observed in carriers of polymorphic *MMP14* rs1042703 allele when compared to noncarriers, but only after adjustment for histological type, weight loss, and performance status (HR = 1.44, 95% CI = 1.01–2.03, *p* = 0.042). Additionally, carriers of two polymorphic *MMP2* rs243849 alleles tended to have shorter TTP after adjustment for clinical parameters (HR = 2.16, 95% CI = 1.02–4.55, *p* = 0.043).

### 3.4. Overall Survival Analysis

**Figure 1:**
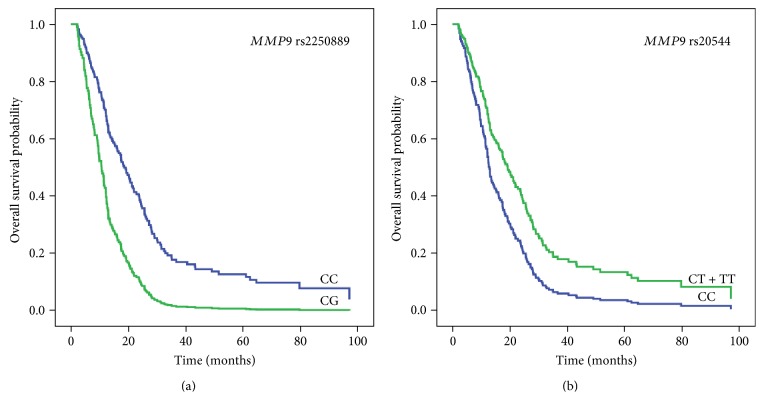
The influence of *MMP9* rs2250889 CC and CG genotypes (a) and *MMP9* rs20544 CC and CT+TT genotypes (b) on overall survival of malignant pleural mesothelioma patients.

The results of OS analysis are shown in Table 3 (analysis adjusted for clinical variables) and Supplementary Table 1 (unadjusted analysis). In multivariable analysis, histological type and performance status were significantly associated with OS. Carriers of polymorphic *MMP9* rs2250889 allele had shorter overall survival (OS) compared to noncarriers (OS 9.23 versus 19.10 months, *p* = 0.002, HR = 2.39, 95% CI = 1.37–4.18, Figure 1(a)). The association was significant also after adjustment for histological type and performance status (HR = 2.52, 95% CI = 1.42–4.46, *p* = 0.002).

Again, carriers of at least one polymorphic *MMP9 rs20544* had longer OS compared to noncarriers (OS 19.3 versus 13.5 months, *p* = 0.014, HR = 0.61, 95% CI = 0.41–0.90, Figure 1(b)). When adjusted for histological type and performance status, the association remained nominally significant (HR = 0.63, 95% CI = 0.42–0.95, *p* = 0.025).

Carriers of two polymorphic alleles *MMP14* rs1042703 had shorter OS compared to the carriers of the wild-type alleles (OS 12.7 versus 17.5 months, *p* = 0.043, HR = 1.92, 95% CI = 1.02–3.06). *MMP14* rs1042703 remained nominally significantly associated with OS (HR = 2.14, 95% CI = 1.12–4.0, *p* = 0.020) after the adjustment for histological type and performance status.

### 3.5. Haplotype Analysis

As two SNPs in *MMP9* were associated with survival, we also performed a haplotype analysis (Table 4). Six different haplotypes covered all the variability in *MMP9*. Polymorphic *MMP9* rs2250889 allele that was associated with survival in single SNP analysis was present on two rare *MMP9* haplotypes, AGGC and AGAC, that were both associated with significantly shorter TTP and OS.

## 4. Discussion

This study investigated the influence of *MMP2, MMP9*, and *MMP14* gene polymorphisms on time to progression and overall survival in mesothelioma patients. Two of the investigated *MMP9* SNPs, rs2250889 and rs20544, had significant but opposite effects on TTP and OS in our patient population. Additionally, nonsynonymous *MMP14* rs1042703 genotype was associated with shorter survival in MPM patients.

*MMP9* rs2250889 polymorphic genotype (c.1721C>G) was strongly and statistically significantly associated with both lower TTP and OS in our study. This polymorphism is nonsynonymous, leading to the p.Arg574Pro substitution, although the function prediction also indicated a possibility that it may influence splicing. As the biological functions of MMP9 in maintenance of tumor stem cells and metastatic niches are well established, there has been a lot of research interest in the *MMP9* gene and its SNPs [26]. We selected *MMP9* SNPs for genotyping in our study after reviewing the literature of more common cancers, such as bladder cancer, where a large meta-analysis was written considering many genetically diverse populations. The meta-analysis included two of our selected SNPs in the MMP9 gene, with varying correlation to clinical data, such as disease progression and overall survival [20].

One of the previous studies in *MMP9* SNPs showed that the risk for developing metastatic lung cancer is higher in heterozygous (p.574 Pro/Arg) and homozygous (p.574 Pro/Pro) carriers of rs2250889 polymorphism compared to noncarriers. In the study that included 744 patients with lung cancer and 747 cancer-free controls from Southeastern Chinese population, subjects with the rs2250889 encoded heterozygous p.574 Pro/Arg and homozygous p.574 Pro/Pro genotypes had 1.46-fold (95% CI = 0.94–2.26) and 1.69-fold elevated risk (95% CI = 1.10–2.60), respectively, compared to subjects with p.574Arg/Arg genotype [27]. Subsequent studies that investigated the prognostic role of *MMP9* rs2250889 in 200 nasopharyngeal carcinoma patients showed increased death risk (HR = 2.287, 95% CI = 1.400–3.735) in subjects with *MMP9* rs2250889 encoded p.574Pro/Pro and p.574Pro/Arg genotypes compared to p.574Arg/Arg genotype [28].

In addition to the abovementioned role of MMP9 in local tumor progression and metastasis, it also has a tumor-suppressing function of producing endogenous angiogenesis inhibitors, promoting inflammatory antitumour activity and inducing apoptosis [26]. This dual biological function could also partially explain the beneficial effect of *MMP9* rs25044 on TTP and OS observed in our study. The function prediction analysis suggested a role of *MMP9* rs25044 in differential miRNA binding. So far, there have been 41 SNP-specific miRNAs identified that target *MMP9* SNPs. miRNAs work with exquisite specificity: they distinguish a target from a nontarget based on a single nucleotide mismatch in the core nucleotide domain leading to translational inhibition and mRNA destabilization with a consequent reduction in the protein levels [29]. The proposed rs25044-miRNA interaction could have a putative protective effect and thus influence survival in MPM patients. Epidemiologic studies in solid cancer (breast, colon, and lung) that investigated selected SNP-miRNA interactions showed either decreased or increased cancer risk [30].

*MMP14* rs1042703 had nominally significant influence on TTP in our study, but only after adjustment for weight loss, histological type, and performance status. The *MMP14* rs1042703 is a nonsynonymous SNP leading to amino acid substitution (p.Pro8Ser) and can thus influence an individual's phenotype. A possible role of *MMP14* rs1042703 as a biomarker in hepatocellular carcinoma (HCC) was investigated in a Taiwanese study with rs1042703 CC genotype resulting in lower *MMP14* expression and lower risk of acquiring HCC [31]. The potentially altered protein structure and phenotype could have a deleterious effect in MPM patients, as suggested by our study.

In our study, we have also investigated *MMP2* SNPs (rs243865, rs243849, and rs7201) that failed to support any statistically significant association with the TTP and OS. The function prediction analysis showed that rs243865 may alter the transcription factors' binding site. This SNP was reported to significantly increase the risk of osteosarcoma in a Chinese Han population. Both heterozygous rs243865 CT and homozygous TT genotype were associated with significantly increased risk for osteosarcoma (OR = 1.86, 95% CI = 1.18–4.22, *p* = 0.014 and OR = 1.92, 95% CI = 1.21–3.52, *p* = 0.028, resp.) [32]. On the other hand, rs243849 may influence splicing, but was, thus far, investigated in a prostate cancer population of 1817 African American men, where the results showed an increased risk of disease aggressiveness (OR = 1.44; *p* = 0.04) in Stage 3 for the T allele of rs243849 [33]. Lastly, rs7201 may lead to differential miRNA binding, and its potential role was investigated in a nasopharyngeal (NC) carcinoma study. MiR-151 correlation to rs7201 was investigated but did not have any statistically significant influence on NC risk [34]. Additionally, data from the Genomic Data Commons Data Portal [35] show that *MMP2* can sometimes be mutated in MPM patients, suggesting further studies of MMP genetic variability could contribute to our understanding of the disease.

The cited studies that investigated the role of our selected SNPs included a limited number of patients and were not setup as genome-wide association studies (GWAS). However, an Italian-based GWAS study that included 407 patients and 389 controls found that *MMP14*, among other genes, could be as a risk factor for MPM. They proposed that there is dysregulation of the *MMP14* in MPM, but there was not any additional data on patient survival or disease progression [36]. Further studies are therefore needed regarding the role of MMP SNPs in MPM.

Our study brings novel interesting findings; however, it has a few limitations, due to the low size number and the fact that we did not perform a GWAS and/or a replication study. As MPM is very rare, the results should be validated in an independent population in the future.

In conclusion, we believe that selected *MMP* SNPs could be valuable prognostic biomarkers in MPM. We also believe that the presented paper has opened the gate in performing further genetic studies on metalloproteases in this deadly disease. Their role may become even more important with the development of new treatment options, such as immunotherapy and targeted therapy, where there is a need for better and more accessible biomarkers.

## Supplementary Material

Supplementary table 1: The influence of MMP genotypes on time to progression and overall survival in MPM patients.

## Figures and Tables

**Table 1:** Patients' characteristics (*N* = 199).

Characteristic		*N* (%)
Gender	Male	151 (75.9)
	Female	48 (24.1)

Age	Median (25%–75%)	66 (58–72)

Stage	I	13 (6.5)
	II	53 (26.6)
	III	61 (30.7)
	IV	54 (27.1)
	Peritoneal	18 (9.0)

Histological type	Epithelioid	143 (71.9)
	Biphasic	25 (12.6)
	Sarcomatoid	21 (10.6)
	Not characterized	10 (5.0)

ECOG performance status	0	10 (5.0)
	1	95 (47.7)
	2	84 (42.2)
	3	10 (5.0)

C-reactive protein	Median (25%–75%)	23 (10–68.3) [29]

Asbestos exposure	Not exposed	43 (22.3) [6]
	Exposed	150 (77.7)

Smoking	Nonsmokers	95 (49.0) [5]
	Smokers	99 (51.0)

Type of chemotherapy	Gemcitabine/cisplatin	123 (61.8)
	Pemetrexed/cisplatin	48 (24.1)
	Other	11 (5.5)
	None	17 (8.6)

Response rate	CR	7 (4.1) [27]
	PR	57 (33.1)
	SD	86 (50.0)
	PD	22 (12.8)

Time to progression	Median (25%–75%)	7.67 (5.27–13.80)

Overall survival	Median (25%–75%)	16.30 (9.07–26.80)

Follow-up	Median (25%–75%)	69.67 (22.00–81.53)

Numbers in square brackets denote the number of patients with missing data. CR: complete response; ECOG: Eastern Cooperative Oncology Group; PD: progressive disease; PR: partial response; SD: stable disease.

**Table 2:** Genotype frequencies of investigated polymorphisms.

Gene	SNP		Genotype	*N* (%)	Predicted function^∗^
*MMP2*	rs243865	c.-1306C>T	CC	130 (65.3)	May influence binding of transcription factors, may alter chromatin states
			CT	66 (33.2)	
			TT	3 (1.5)	
	rs243849	c.999C>T, p.Asp333=	CC	140 (70.4)	May influence splicing
			CT	51 (25.6)	
			TT	8 (4.0)	
	rs7201	c.^∗^260A>C	AA	67 (33.7)	Differential miRNA binding may alter regulatory motifs and tissue-specific gene expression
			AC	96 (48.2)	
			CC	36 (18.1)	

*MMP9*	rs17576	c.836A>G, p.Gln279Arg	AA	80 (40.6) [2]	Nonsynonymous may change protein function or structure, may influence splicing
			AG	98 (49.7)	
			GG	19 (9.6)	
	rs2250889	c.1721C>G, p.Arg574Pro	CC	181 (91.0)	Nonsynonymous may influence splicing
			CG	18 (9.0)	
			GG	0 (0.0)	
	rs17577	c.2003G>A, p.Arg668Gln	GG	141 (71.6) [2]	Nonsynonymous may influence splicing
			GA	53 (26.9)	
			AA	3 (1.5)	
	rs20544	c.^∗^3C>T	CC	37 (18.7) [1]	Differential miRNA binding may alter regulatory motifs and tissue-specific gene expression
			CT	101 (51.0)	
			TT	60 (30.3)	

*MMP14*	rs1042703	c.22T>C, p.Pro8Ser	TT	130 (67.0) [5]	Nonsynonymous
			TC	53 (27.3)	
			CC	11 (5.7)	
	rs1042704	c.817G>A, p.Asp273Asn	GG	135 (67.8)	Nonsynonymous may influence splicing
			GA	54 (27.1)	
			AA	10 (5.0)	
	rs743257	c.^∗^83C>T	CC	50 (25.1)	Differential miRNA binding may alter chromatin states and regulatory motifs
			CT	86 (43.2)	
			TT	63 (31.7)	

Numbers in square brackets denote the number of patients with missing data. ^∗^Evaluated using SNP function prediction [22]; HaploReg [23] and GTEx [24].

**Table 3:** The influence of *MMP* genotypes on time to progression and overall survival in MPM patients, adjusted for clinical variables.

Gene	Genotype	Time to progression	HR (95% CI)	*p*	Overall survival	HR (95% CI)	*p*
		Median (25%–75%)			Median (25%–75%)		
*MMP2*							
rs243865	CC	8.83 (6.43–15.03)	Ref.		17.43 (10.60–27.27)	Ref.	
	CT	10.17 (6.00–16.33)	0.88 (0.63–1.22)	0.437	17.47 (9.47–31.17)	0.96 (0.68–1.36)	0.826
	TT	14.97 (8.20–44.57)	0.54 (0.17–1.75)	0.305	30.00 (22.03–30.00)	0.44 (0.11–1.81)	0.255
	CT + TT	10.20 (6.27–16.33)	0.85 (0.61–1.18)	0.334	18.07 (9.63–31.17)	0.92 (0.66–1.30)	0.653
rs243849	CC	9.87 (6.13–16.67)	Ref.		19.10 (9.83–28.13)	Ref.	
	CT	8.67 (6.67–14.30)	1.30 (0.91–1.86)	0.144	16.23 (10.80–29.03)	1.26 (0.86–1.85)	0.227
	TT	7.33 (3.27–11.80)	**2.16 (1.02–4.55)**	**0.043**	17.63 (7.33–36.73)	1.18 (0.51–2.72)	0.698
	CT + TT	8.67 (6.60–14.30)	1.38 (0.98–1.94)	0.064	16.63 (10.80–30.27)	1.25 (0.87–1.80)	0.225
rs7201	AA	9.27 (6.37–16.93)	Ref.		16.23 (10.63–28.03)	Ref.	
	AC	10.00 (6.73–15.03)	0.87 (0.61–1.24)	0.441	19.30 (10.80–30.00)	0.89 (0.62–1.29)	0.547
	CC	8.00 (5.50–12.63)	1.15 (0.73–1.80)	0.551	14.20 (9.23–26.17)	1.20 (0.74–1.95)	0.467
	AC + CC	9.80 (6.43–14.57)	0.93 (0.67–1.30)	0.684	18.07 (9.63–29.03)	0.96 (0.67–1.36)	0.803

*MMP9*							
rs17576	AA	9.27 (6.13–13.53)	Ref.		13.57 (9.83–23.90)	Ref.	
	AG	10.73 (6.80–18.47)	0.84 (0.60–1.17)	0.303	21.20 (12.17–32.53)	0.72 (0.50–1.03)	0.070
	GG	8.50 (6.00–16.67)	1.17 (0.69–1.98)	0.558	16.63 (8.27–26.60)	0.96 (0.54–1.68)	0.881
	AG + GG	10.00 (6.67–16.90)	0.89 (0.65–1.23)	0.478	19.30 (11.33–31.17)	0.76 (0.54–1.06)	0.106
rs2250889	CC	10.03 (6.73–16.67)	Ref.		19.10 (11.33–30.00)	Ref.	
	CG	6.07 (4.07–7.90)	**2.32 (1.34–4.03)**	**0.003**	9.23 (4.53–14.20)	**2.52 (1.42–4.46)**	**0.002**
rs17577	GG	10.00 (6.60–16.33)	Ref.		18.07 (10.60–28.30)	Ref.	
	GA	8.33 (5.50–14.57)	1.20 (0.84–1.72)	0.319	17.63 (9.47–32.87)	0.82 (0.56–1.20)	0.311
	AA	8.83 (8.00–19.43)	1.02 (0.32–3.25)	0.977	13.50 (9.97–25.93)	1.58 (0.49–5.04)	0.444
	GA + AA	8.50 (5.53–14.57)	1.19 (0.84–1.68)	0.339	17.63 (9.63–32.53)	0.85 (0.59–1.24)	0.403
rs20544	CC	7.53 (5.53–11.53)	Ref.		13.50 (8.13–21.20)	Ref.	
	CT	10.93 (7.37–19.97)	**0.60 (0.40–0.90)**	**0.014**	20.67 (11.33–32.53)	**0.59 (0.39–0.91)**	**0.018**
	TT	9.40 (6.27–14.30)	0.67 (0.43–1.03)	0.069	15.40 (10.80–25.67)	0.70 (0.44–1.11)	0.132
	CT + TT	10.13 (6.73–16.20)	**0.62 (0.43–0.92)**	**0.016**	19.30 (10.80–31.17)	**0.63 (0.42–0.95)**	**0.025**

*MMP14*							
rs1042703	TT	9.27 (6.67–16.33)	Ref.		17.50 (9.97–29.13)	Ref.	
	TC	10.17 (6.27–14.53)	1.36 (0.94–1.96)	0.100	19.30 (9.47–29.03)	1.25 (0.87–1.81)	0.229
	CC	8.70 (5.13–16.20)	**2.09 (1.06–4.12)**	**0.032**	12.70 (7.07–20.60)	**2.14 (1.12–4.06)**	**0.020**
	TC + CC	10.17 (6.03–14.97)	**1.44 (1.01–2.03)**	**0.042**	17.63 (8.30–28.30)	1.36 (0.97–1.92)	0.076
rs1042704	GG	9.80 (6.73–14.90)	Ref.		17.50 (10.80–31.17)	Ref.	
	GA	8.30 (5.70–16.20)	1.05 (0.74–1.49)	0.780	17.47 (9.10–24.23)	1.40 (0.98–2.00)	0.068
	AA	7.87 (6.00–16.90)	0.90 (0.45–1.82)	0.777	20.27 (13.57–25.93)	1.22 (0.59–2.53)	0.598
	GA + AA	8.30 (5.93–16.90)	1.03 (0.74–1.42)	0.881	18.07 (9.63–24.57)	1.37 (0.97–1.92)	0.072
rs743257	CC	9.87 (7.07–14.07)	Ref.		13.50 (9.97–27.27)	Ref.	
	CT	9.40 (5.97–19.43)	1.08 (0.72–1.64)	0.700	15.40 (6.80–28.13)	0.98 (0.65–1.49)	0.931
	TT	9.70 (6.60–14.53)	1.15 (0.75–1.76)	0.535	20.17 (12.53–31.17)	0.87 (0.56–1.36)	0.544
	CT + TT	9.43 (6.07–16.33)	1.11 (0.76–1.63)	0.588	19.30 (9.83–30.27)	0.93 (0.63–1.37)	0.722

**Table 4:** The influence of *MMP9* haplotypes on time to progression and overall survival.

Haplotype	Estimated frequency	TTP HR (95% CI)	*p*	OS HR (95% CI)	*p*
ACGT	0.560	Reference		Reference	
GCGC	0.214	0.98 (0.74–1.29)	0.877	0.97 (0.73–1.30)	0.853
GCAC	0.129	1.13 (0.81–1.58)	0.460	0.96 (0.68–1.37)	0.830
ACGC	0.053	1.33 (0.81–2.18)	0.261	1.85 (1.12–3.05)	**0.016**
AGGC	0.024	2.14 (1.03–4.44)	**0.042**	2.07 (1.02–4.22)	**0.045**
AGAC	0.020	2.93 (1.30–6.60)	**0.010**	3.38 (1.33–8.54)	**0.010**

The SNPs are ordered from the 5′- to 3′-end as follows: rs17576, rs2250889, rs17577, and rs20544.
