# Supplementary material for: Matrix Metalloproteinases Polymorphisms as Prognostic Biomarkers in Malignant Pleural Mesothelioma
Source: Dis Markers. 2017 Sep 12;2017:8069529. doi: 10.1155/2017/8069529 (PMC5613362; doi:10.1155/2017/8069529)
Supplement: Supplementary file 1 — Supplementary table 1: The influence of MMP genotypes on time to progression and overall survival in MPM patients. [file 8069529.f1.pdf]

Supplementary table 1: The influence of *MMP* genotypes on time to progression and overall survival in MPM patients.

**Gene**	**Genotype**	**Time to progression**			**Overall survival**		
		Median (25%-75%)	HR (95% CI)	P	Median (25%-75%)	HR (95% CI)	P
MMP2							
rs243865	CC	8.83 (6.43-15.03)	Ref.		17.43 (10.60-27.27)	Ref.	
	CT	10.17 (6.00-16.33)	0.91 (0.66-1.25)	0.547	17.47 (9.47-31.17)	0.91 (0.65-1.28)	0.605
	TT	14.97 (8.20-44.57)	0.55 (0.17-1.74)	0.309	30.00 (22.03-30.00)	0.38 (0.09-1.54)	0.173
	CT+TT	10.20 (6.27-16.33)	0.88 (0.64-1.2)	0.418	18.07 (9.63-31.17)	0.87 (0.62-1.22)	0.416
rs243849	CC	9.87 (6.13-16.67)	Ref.		19.10 (9.83-28.13)	Ref.	
	CT	8.67 (6.67-14.30)	1.05 (0.75-1.49)	0.769	16.23 (10.80-29.03)	1.05 (0.72-1.52)	0.795
	TT	7.33 (3.27-11.80)	1.34 (0.65-2.75)	0.424	17.63 (7.33-36.73)	0.89 (0.39-2.02)	0.774
	CT+TT	8.67 (6.60-14.30)	1.09 (0.79-1.51)	0.610	16.63 (10.80-30.27)	1.02 (0.72-1.46)	0.892
rs7201	AA	9.27 (6.37-16.93)	Ref.		16.23 (10.63-28.03)	Ref.	
	AC	10.00 (6.73-15.03)	0.97 (0.69-1.36)	0.867	19.30 (10.80-30.00)	0.95 (0.66-1.36)	0.760
	CC	8.00	1.15	0.535	14.20	1.19	0.453

		(5.50-12.63)	(0.74-1.78)		(9.23-26.17)	(0.75-1.90)	
	AC+CC	9.80 (6.43-14.57)	1.02 (0.74-1.4)	0.924	18.07 (9.63-29.03)	1.00 (0.71-1.41)	0.980
MMP9							
rs17576	AA	9.27 (6.13-13.53)	Ref.		13.57 (9.83-23.90)	Ref.	
	AG	10.73 (6.80-18.47)	0.75 (0.54-1.04)	0.083	21.20 (12.17-32.53)	**0.70 (0.49-0.98)**	**0.041**
	GG	8.50 (6.00-16.67)	1.17 (0.69-1.97)	0.557	16.63 (8.27-26.60)	1.04 (0.60-1.81)	0.884
	AG+GG	10.00 (6.67-16.90)	0.81 (0.59-1.1)	0.172	19.30 (11.33-31.17)	0.74 (0.53-1.03)	0.079
rs2250889	CC	10.03 (6.73-16.67)	Ref.		19.10 (11.33-30.00)	Ref.	
	CG	6.07 (4.07-7.90)	**2.45 (1.45-4.14)**	**0.001**	9.23 (4.53-14.20)	**2.39 (1.37-4.18)**	**0.002**
rs17577	GG	10.00 (6.60-16.33)	Ref.		18.07 (10.60-28.30)	Ref.	
	GA	8.33 (5.50-14.57)	1.19 (0.84-1.67)	0.322	17.63 (9.47-32.87)	0.91 (0.62-1.32)	0.609
	AA	8.83 (8.00-19.43)	1.15 (0.37-3.63)	0.809	13.50 (9.97-25.93)	1.51 (0.48-4.78)	0.481
	GA+AA	8.50 (5.53-14.57)	1.19 (0.85-1.66)	0.317	17.63 (9.63-32.53)	0.93 (0.65-1.35)	0.717
rs20544	CC	7.53 (5.53-11.53)	Ref.		13.50 (8.13-21.20)	Ref.	
	CT	10.93	**0.57**	**0.007**	20.67	**0.56**	**0.007**

		(7.37-19.97)	**(0.38-0.86)**		(11.33-32.53)	**(0.37-0.85)**	
	TT	9.40 (6.27-14.30)	0.72 (0.47-1.11)	0.139	15.40 (10.80-25.67)	0.70 (0.44-1.11)	0.133
	CT+TT	10.13 (6.73-16.20)	**0.63 (0.43-0.91)**	**0.015**	19.30 (10.80-31.17)	**0.61 (0.41-0.90)**	**0.014**
MMP14							
rs1042703	TT	9.27 (6.67-16.33)	Ref.		17.50 (9.97-29.13)	Ref.	
	TC	10.17 (6.27-14.53)	1.03 (0.73-1.45)	0.879	19.30 (9.47-29.03)	1.10 (0.77-1.57)	0.594
	CC	8.70 (5.13-16.20)	1.28 (0.68-2.38)	0.442	12.70 (7.07-20.60)	**1.92 (1.02-3.61)**	**0.043**
	TC+CC	10.17 (6.03-14.97)	1.07 (0.77-1.47)	0.695	17.63 (8.30-28.30)	1.20 (0.86-1.67)	0.284
rs1042704	GG	9.80 (6.73-14.90)	Ref.		17.50 (10.80-31.17)	Ref.	
	GA	8.30 (5.70-16.20)	1.08 (0.77-1.52)	0.655	17.47 (9.10-24.23)	1.35 (0.94-1.92)	0.101
	AA	7.87 (6.00-16.90)	1.01 (0.53-1.94)	0.971	20.27 (13.57-25.93)	0.95 (0.46-1.95)	0.884
	GA+AA	8.30 (5.93-16.90)	1.07 (0.78-1.47)	0.686	18.07 (9.63-24.57)	1.27 (0.90-1.77)	0.170
rs743257	CC	9.87 (7.07-14.07)	Ref.		13.50 (9.97-27.27)	Ref.	
	CT	9.40	0.85	0.416	15.40	0.87	0.512

		(5.97-19.43)	(0.58-1.26)		(6.80-28.13)	(0.58-1.31)	
	TT	9.70 (6.60-14.53)	0.97 (0.65-1.46)	0.901	20.17 (12.53-31.17)	0.79 (0.52-1.22)	0.287
	CT+TT	9.43 (6.07-16.33)	0.9 (0.63-1.29)	0.575	19.30 (9.83-30.27)	0.84 (0.58-1.21)	0.346
